# Contribution of a heparin-binding haemagglutinin interferon-gamma release assay to the detection of *Mycobacterium tuberculosis* infection in HIV-infected patients: comparison with the tuberculin skin test and the QuantiFERON®-TB Gold In-tube

**DOI:** 10.1186/s12879-015-0796-0

**Published:** 2015-02-14

**Authors:** Chloé Wyndham-Thomas, Violette Dirix, Kinda Schepers, Charlotte Martin, Marc Hildebrand, Jean-Christophe Goffard, Fanny Domont, Myriam Libin, Marc Loyens, Camille Locht, Jean-Paul Van Vooren, Françoise Mascart

**Affiliations:** Laboratory of Vaccinology and Mucosal Immunology, Université Libre de Bruxelles, Brussels, Belgium; Immunodeficiency unit, Hôpital Erasme, Brussels, Belgium; Infectious Disease, CHU Saint-Pierre, Brussels, Belgium; Infectious disease unit, IRIS SUD hospitals, Brussels, Belgium; INSERM U1019, Lille, France; CNRS UMR8204, Lille, France; Université Lille Nord de France, Lille, France; Center for Infection and Immunity of Lille, Institut Pasteur de Lille, Lille, France; Immunobiology Clinic, Hôpital Erasme, Brussels, Belgium

**Keywords:** Active tuberculosis, Heparin-binding haemagglutinin, Human, Human immunodeficiency virus, Interferon-gamma release assay, Latent tuberculosis, Multiplex, *Mycobacterium tuberculosis*, QuantiFERON®-TB Gold In-Tube, Tuberculin skin test

## Abstract

**Background:**

The screening and treatment of latent tuberculosis (TB) infection reduces the risk of progression to active disease and is currently recommended for HIV-infected patients. The aim of this study is to evaluate, in a low TB incidence setting, the potential contribution of an interferon-gamma release assay in response to the mycobacterial latency antigen Heparin-Binding Haemagglutinin (HBHA-IGRA), to the detection of *Mycobacterium tuberculosis* infection in HIV-infected patients.

**Methods:**

Treatment-naïve HIV-infected adults were recruited from 4 Brussels-based hospitals. Subjects underwent screening for latent TB using the HBHA-IGRA in parallel to a classical method consisting of medical history, chest X-ray, tuberculin skin test (TST) and QuantiFERON®-TB Gold In-Tube (QFT-GIT). Prospective clinical and biological follow-up ensued, with repeated testing with HBHA-IGRA. A group of HIV-infected patients with clinical suspicion of active TB was also recruited and tested with the HBHA-IGRA. Multiplex analysis was performed on the culture supernatants of this in-house assay to identify test read-outs alternative to interferon-gamma that could increase the sensitivity of the test.

**Results:**

Among 48 candidates enrolled for screening, 9 were identified with latent TB by TST and/or QFT-GIT results. Four of these 9 patients and an additional 3 screened positive with the HBHA-IGRA. This in-house assay identified all the patients that were positive for the TST and showed the best concordance with the presence of a *M. tuberculosis* exposure risk. During follow-up (median 14 months) no case of active TB was reported and HBHA-IGRA results remained globally constant. Fourteen HIV-infected patients with clinical suspicion of active TB were recruited. Active TB was confirmed for 6 of them among which 3 were HBHA-IGRA positive, each with very high interferon-gamma concentrations. All patients for whom active TB was finally excluded, including 2 non-tubercular mycobacterial infections, had negative HBHA-IGRA results. Multiplex analysis confirmed interferon-gamma as the best read-out.

**Conclusions:**

The HBHA-IGRA appears complementary to the QuantiFERON®-TB Gold In-Tube for the screening of latent TB in HIV-infected patients. Large-scale studies are necessary to determine whether this combination offers sufficient sensitivity to dismiss TST, as suggested by our results. Furthermore, HBHA-IGRA may help in the diagnosis work-up of clinical suspicions of active TB.

## Background

In HIV-infected subjects the risk of reactivation of a latent tuberculosis infection (LTBI) reaches 10% per year, and the overall risk of developing active tuberculosis (TB) is 21 to 34 times greater than for non HIV-infected individuals [1,2]. Although this risk decreases with treatment by combination antiretroviral therapy (cART), it still remains twice that of the general population [3]. Consequently, TB remains the main cause of death in HIV-infected patients. In 2012, 13% of the 8.6 million new cases of TB reported worldwide were diagnosed in patients living with HIV and 320,000 people died of HIV-associated TB [4]. In response to this threat, prevention of TB by the screening and treatment of LTBI in HIV-infected patients is now recommended by international guidelines and promoted by the World Health Organisation’s “Three I’s” strategy for TB control (“*Intensified case finding, Isoniazid preventive therapy and TB Infection control for people living with HIV”*) [4-6].

The currently available tests used for the detection of LTBI are the tuberculin skin test (TST), the QuantiFERON®-TB Gold In-Tube (QFT-GIT) and the T-SPOT.TB®. The TST measures the *in vivo* delayed hypersensitivity response to mycobacterial antigens contained in Purified Protein Derivative (PPD). The test lacks sensitivity, particularly in HIV-infected subjects, and has a low specificity due to cross-reactivity with the BCG vaccine and non-tubercular mycobacteria [7,8]. The QFT-GIT and the T-SPOT.TB® are T-cell based interferon-gamma-release assays (IGRA) that measure respectively the levels of Interferon-gamma (IFN-γ) released and the number of IFN-γ-producing cells after an *in vitro* stimulation by specific RD-1/RD-11 *Mycobacterium tuberculosis (Mtb)* antigens. These two assays demonstrate a greater specificity than TST for the diagnosis of LTBI but their sensitivities remain insufficient [9,10]. Discordant results between the 3 tests are frequent in HIV-infected patients, even in low BCG vaccination settings [11], and combining TST and an IGRA is therefore encouraged to increase the sensitivity of screening [5,6].

Various strategies to discover superior LTBI screening tools are being explored, including the development of IGRA in response to alternative *Mtb* antigens not present in QFT-GIT and T-SPOT.TB®. A potential candidate is the Heparin-Binding Haemagglutinin (HBHA), a methylated *Mtb* protein regarded as a latency antigen. Indeed, most LTBI subjects show high levels of IFN-γ secretion by their peripheral lymphocytes upon stimulation with HBHA. The levels of IFN-γ reached are significantly higher than those recorded both in subjects free of *Mtb* infection and in patients with active TB [12,13]. An in-house IGRA based on HBHA (HBHA-IGRA) has been shown to be a promising LTBI screening tool, both in immune-competent adults and in haemodialysed patients [13,14]. In HIV-infected patients, the only available results concerning the IFN-γ response to HBHA are those published by Loxton *et al*., who tested the antigenic response with a whole blood assay in 4 subjects living in South Africa [15]. The aim of the present study is to evaluate, in a low TB incidence setting, the potential contribution of the HBHA-IGRA to the screening of LTBI in HIV-infected patients.

## Methods

### Ethics statement

The protocols for this study (P2011/311 and P2011/113) received approval from the leading ethics committee “ULB - Hôpital Erasme” (aggregation number OMO21) and all participants signed an informed consent.

### Study setting

The study was set in Belgium, a low TB incidence country of less than 10 cases/100,000 inhabitants, and more precisely in its capital Brussels that reports a TB incidence of 27.4/100,000 inhabitants [16]. Patient enrolment took place between December 2011 and December 2012, and clinical follow-up was pursued until November 2013.

### Study outline

Treatment-naïve HIV-infected adults (≥18 years old) were recruited from 4 different hospitals. Exclusion criteria were pregnancy, breast-feeding and anti-TB treatment within the past 6 months. The subjects enrolled underwent LTBI screening at baseline with an HBHA-IGRA in parallel to a classical approach consisting of medical history, chest X-ray, TST and QFT-GIT. Prospective clinical follow-up ensued and all relevant events documented: initiation of LTBI treatment, initiation of cART, novel *Mtb* exposure risk factors and development of active TB or TB-associated immune reconstitution inflammatory syndrome (TB-IRIS). The HBHA-IGRA was repeated during the first year of follow-up at the rhythm established by the treating physician for the evaluation of the patient’s HIV-infection parameters.

A group of HIV-positive adults with clinical suspicion of active TB were also recruited and tested with HBHA-IGRA. The objective was to evaluate whether the HBHA-IGRA results obtained in the course of active TB differ from those obtained in LTBI. Indeed, a relative discrimination between TB and LTBI by an HBHA-IGRA performed on PBMC has been described in HIV uninfected persons but whether this asset persists in HIV-infected patients remains unknown [13]. For this group of patients, blood was sampled for the HBHA-IGRA prior or within 5 days of anti-TB treatment and, if TB was confirmed, repeated after at least 1 month of therapy. Diagnosis of TB was based either on microbiological proof or high clinical suspicion with favourable response to anti-TB treatment. When applicable, the infections were qualified as “unmasking TB-IRIS”, “paradoxal TB-IRIS” or “ART-associated” using the International Network for the Study of HIV-associated IRIS (INSHI) case definitions [17]. Again, relevant clinical events arising during treatment were documented.

For all patients CD4^+^ T cell counts, CD4^+^ percentages, CD4^+^/CD8^+^ ratios and viral load performed within 1 month of the HBHA-IGRA were recorded at baseline and throughout follow-up.

Finally, various chemokines and cytokines were quantified by the multiplex technology in the culture supernatants of a selection of QTF-GIT and HBHA-IGRA with the aim of identifying complementary test read-outs to IFN-γ.

## Classical LTBI screening: medical history, chest X-ray, TST and QFT-GIT

Medical history was taken using a questionnaire on general epidemiological data, BCG vaccination status, risk factors for *Mtb* exposure and for LTBI reactivation. The questionnaire was completed by participating infectious disease specialists. Chest X-ray was obtained with postero-anterior and lateral views and interpreted in each hospital by a local radiologist. TST was performed by the intradermal injection of 2 units of PPD RT23 (Statens Serum Institute, Denmark) and indurations were evaluated 72 hours later by the aforementioned infectious disease specialists. The cut-off of positivity used was a diameter of induration ≥5 mm, as is currently recommended for HIV-infected patients [6,18]. QFT-GIT was the commercialized IGRA used (Qiagen, Venlo, The Netherlands). Analysis and interpretation was performed by the laboratory of Immunobiology of the Hôpital Erasme, following the manufacturer’s instructions. Blood was sampled for QFT-GIT, as for the in-house HBHA-IGRA, on the first day of TST or exceptionally within 3 days to avoid a boosting effect of the PPD injection [19].

## HBHA-IGRA

The HBHA-IGRA was performed as previously described [20]. Briefly, peripheral blood mononuclear cells (PBMC) were purified from fresh blood samples. Four million cells were then distributed equally into 4 tubes to allow simultaneous stimulation with (1) 2 μg/ml of native HBHA (purified as previously described [21]), (2) 4 μg/ml of PPD (Statens serum institute, Copenhagen, Denmark) as a signal of previous mycobacterial exposure, (3) 0.5 μg/ml of superantigen SEB (Sigma Aldrich, Bornem, Belgium) as a positive control or (4) medium alone as a negative control. In each tube, 1 ng/ml of Interleukin-7 (R&D Systems Europe, Abington, UK) was added, a technique that has recently shown to increase the sensitivity of the assay without modifying its specificity [20]. The stimulated cells were then incubated in a RPMI 1640 medium (Lonza, Verviers, Belgium) enriched as detailed elsewhere [22]. Culture supernatants were harvested after 24 hours and stored at −20 C. The IFN-γ concentrations in the supernatants were measured by classical sandwich ELISA (Elisa IFN-γ Cytoset, Life technologies, Gent, Belgium). When detectable, the concentrations obtained in the antigen-free condition were subtracted from those obtained with antigen stimulation in order to measure the antigen-specific response. Tests with IFN-γ concentrations below 100 pg/ml in response to SEB were considered indeterminate. The cut-offs applied for the IFN-γ concentrations in response to PPD and HBHA were 200 pg/ml and 50 pg/ml, respectively, as has been defined in immune-competent adults. Positive responses to both PPD and HBHA were necessary for the assay to be considered positive, as this interpretation method has been previously validated and increases the specificity of the test [20].

## Multiplex analysis

For selected subjects, a panel of analytes (IL-6, IL-8, IP-10, MCP-1, MIP-1β, GM-CSF, IL-10, IL-13, IL-17A, IL-2, TNF-α and sCD40L) were quantified in the culture supernatants of their HBHA-IGRA (PPD-, HBHA- and non-stimulated tubes) and QFT-GIT tests (TB Antigen tube and Nil tube) using MILLIPLEX® MAG panels (Merckx Millipore, Brussels, Belgium) following the manufacturers’ instructions. However, culture supernatants were initially diluted using dilution factors specific to each analyte in order to obtain concentrations lying within an interpretable range. Results were analysed with a Bio-Plex® MAGPIX™ Multiplex reader, Bio-Plex Manager™ MP Software and Bio-Plex Manager 6.1 Software (BIO-RAD laboratories, Nazareth Eke, Belgium). If detectable, the analyte concentrations obtained in the antigen-free conditions were subtracted from those obtained with antigen stimulation. To allow statistical analysis with continuous variables, the concentrations below the detection limit were allocated an arbitrary value of 10 pg/ml, representing an undetectable response, whilst results exceeding the assay’s upper limit of detection were attributed the concentration corresponding to this limit.

## Statistical methods

Results were analyzed using GraphPad Prism version 5.04 (GraphPad Software, La Jolla California USA, www.graphpad.com). The Mann–Whitney *U* test and the Kruskall-Wallis test were applied to compare continuous variables between groups. Univariate analysis was performed by Fisher’s exact test and odds ratios for categorical variables. Chi-squared test for trend was applied for ordinal variables. Correlation between continuous variables was assessed using Pearson's product moment coefficient. A p-value <0.05 was considered significant.

## Results

### Population characteristics

Forty-eight treatment-naïve HIV-infected patients were enrolled between December 2011 and December 2012. Demographic characteristics, *Mtb* exposure and LTBI reactivation risk factors, as well as BCG vaccination status and HIV-infection characteristics at baseline are summarized in Table 1.

**Table 1:** **Baseline characteristics of the LTBI screening candidates**

**Demographics**	**Number (n = 48)**
Age; median [range]	38 [22–67]
Gender	
Female	15 (31%)
Male	33 (69%)
Ethnic origin	
Sub Saharan	22 (46%)
Caucasian	18 (38%)
North African	3 (6%)
Asian	3 (6%)
South American	2 (4%)
*Mtb* exposure risk factors	
Presence of at least one major *Mtb* exposure risk factor	23 (48%)
Born in high incidence country^a^	22 (46%)
Arrival in low incidence country ≤ 2 years	4 (8%)^b^
Self-reported close contact with a case of TB	1
Close contact with a case of sputum positive TB	1
Other^c^	5 (10%)
Visitors of endemic countries^d^	14 (29%)
LTBI reactivation risk factors	
Presence of ≥ 1 reactivation risk factor other than HIV	5 (10%)
Diabetes	2 (4%)
Body mass index < 18,5	2 (4%)
Renal insufficiency +/− dialysis	1
Solid tumor	1
Alcoholism	1
Self-reported BCG vaccination status	
Vaccinated	16 (33%)
Not vaccinated	17 (35%)
Unknown	15 (31%)
HIV infection parameters	
HIV-1	47 (98%)
HIV-2	1
Seroconversion ≤ 2 years	9 (19%)^e^
CD4^+^ T-cell count; median [range]	517 [1–1065]
Viral load; median [range]	23242 [<40-1.10^7^]^f^

Units: Age (years); CD4^+^ T-cell count (cells/mm^3^); viral load (copies/ml).

^a^>100 cases of TB/ 100000 inhabitants.

^b^Represents 17% of those born in a high incidence country.

^c^Professional risk, previous incarceration, asylum seeker, homeless.

^d^Concerns the patients born in low incidence country.

^e^Date of HIV seroconversion unknown for 40% of the enrolled subject.s

^f^The patient with an undetectable viral load is an elite controller (HIV-2 infected).

Three patients were lost to follow-up. For the remaining patients, the median clinical follow up time reached 418 days, ranging from 6 to 23 months. cART was initiated in 26 patients at various time points, 15 immediately after enrolment. The median increase in CD4^+^ T-cell counts between baseline values and the final measurement was 113 cells/mm^3^ in treated patients, and 6 patients showed an increase of >200 cells/mm^3^. Isoniazid prophylactic treatment was offered to 3 patients, but, due to non-compliance, completed only by one. Six patients travelled to countries of moderate to high TB incidence (>40 cases/100,000 inhabitants) during follow-up, but no other major risk factor for *Mtb* exposure was recorded. No patients developed active TB.

Fourteen HIV patients with suspected TB were enrolled and are discussed separately in the corresponding paragraph.

### LTBI screening results

Amongst the 48 HIV-infected patients that underwent a screen for LTBI, 23 had at least one major *Mtb* exposure risk factor revealed by the questionnaire (Table 1). The item “visitors of endemic countries” of the questionnaire lacked the precisions required to categorise the travels in terms of *Mtb* exposure risk (duration, conditions etc.) and was therefore classified separately. Seven patients had an LTBI reactivation risk factor in addition to HIV-infection.

Chest X-ray was performed in 46 patients and was found suspicious in 3, with bilateral apical infiltrates in one, right lobe apical infiltrates with fibrotic scars and possible calcified mediastinal lymph node in the second and bi-apical fibrotic scarring with retraction for the third. Complementary Computed Tomography Scan (CT-scan) of the chest was performed in the first 2 patients and the initially described abnormalities were not confirmed. All 3 patients had negative results for the QFT-GIT, TST and HBHA-IGRA.

**Figure 1:**
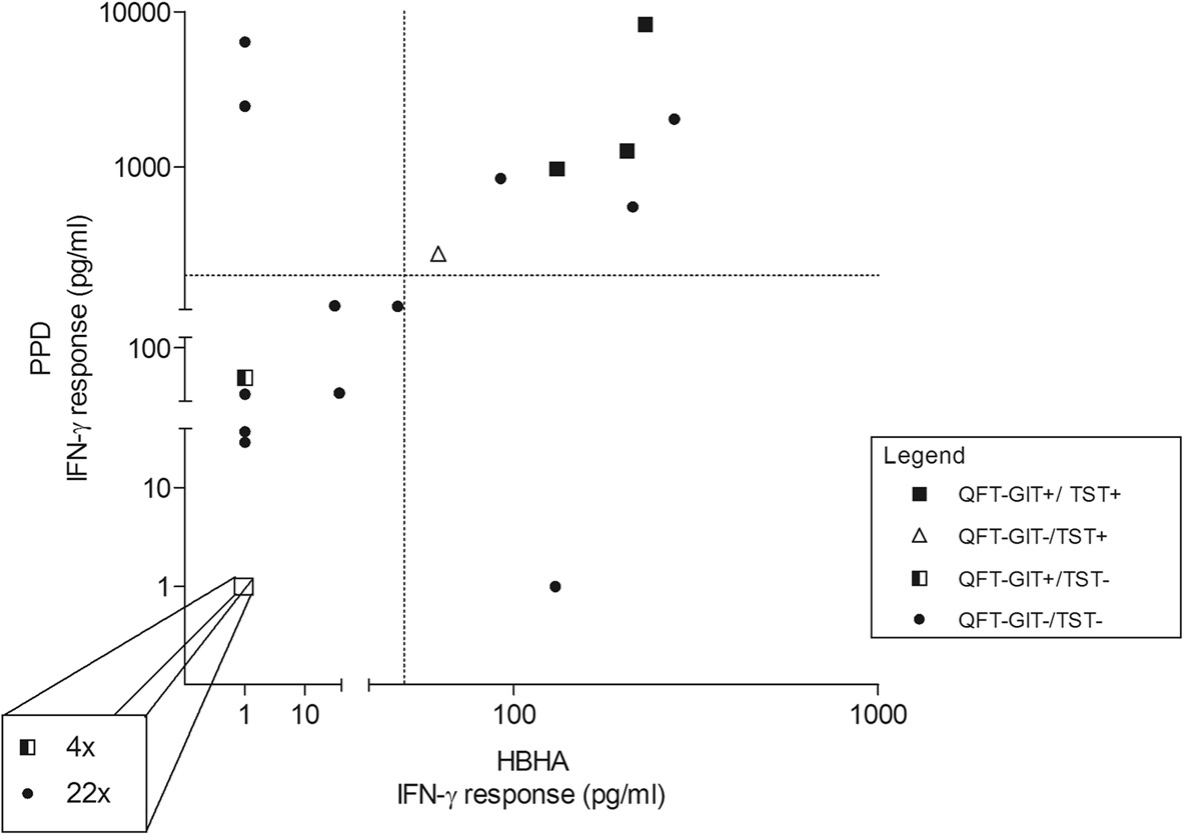
**LTBI screening in treatment-naïve HIV-infected patients: relation between HBHA-IGRA, QFT-GIT and TST result.** Forty-eight treatment naïve HIV-infected subjects underwent screening for latent tuberculosis with 3 different immunological tools: TST, QFT-GIT and HBHA-IGRA. HBHA-IGRA results were interpretable for 43 subjects. The test measures both IFN-γ responses to PPD and to HBHA that must be above or equal to 200 pg/ml and 50 pg/ml respectively for the assay to be considered positive. These cut-offs are represented in the graph as dotted lines. Each point on the graph represents the results of an individual. The format of the point (black square, black dot, white triangle or black and white square) represents the TST and QFT-GIT results obtained for the given individual, as indicated in the legend of the graph. As shown in the magnified square, 26 patients had undetectable IFN-γ levels in response to both PPD and HBHA, including 4 that had QFT-GIT positive tests.

TST results were available for 46 individuals as 2 patients did not return for TST result reading, whilst QFT-GIT and HBHA-IGRA were available for all. HBHA-IGRA, as detailed in the methods, combines PPD- and HBHA-induced IFN-γ responses. Results of this test in relation to TST and QFT-GIT results are shown in Figure 1. At baseline, TST was positive for 4 of the screened subjects (all with an induration diameter ≥10 mm), among which 3 had a concomitant positive QFT-GIT (all with an antigen response >2 IU/ml), and all 4 were positive with the HBHA-IGRA. These 4 patients had reported *Mtb* exposure risks and their respective CD4^+^ T-cell counts went from 98 to 912 cells/mm^3^.

Five patients tested positive only with the QFT-GIT, with antigen responses ranging from 0.82 to 1.98 IU/ml. All these patients had CD4^+^ T-cell counts above 450 cells/mm^3^. Only 2 had a clear *Mtb* exposure risk.

The HBHA-IGRA detected 3 additional patients, one of whom had an indeterminate QFT-GIT result due to an excessive value for the negative control. All 3 subjects were born and lived for more than 20 years in countries with TB incidence above 40 cases/100,000 inhabitants [23], compatible with *Mtb* exposure and potential LTBI. Their CD4^+^ T-cell counts at the time of screening were equal or above 450 cells/mm^3^. Of note, for 5 patients out of the 48 screened, the HBHA-IGRA was indeterminate, one due to a technical error and the 3 others for absence of IFN-γ responses to the positive control.

As shown in Figure 1, two subjects had IFN-γ responses to PPD (above the 200 pg/ml cut-off) but not to HBHA, and all other latent tuberculosis tests negative. These results may be caused by previous exposure to non-tubercular mycobacteria (both grew up in sub-Saharan Africa) or previous BCG-vaccination (recorded in at least one). Inversely, one patient had an IFN-γ response to HBHA but not to PPD, although this result was probably an error as it was no longer found upon repetition of the assay. As expected, all TST positive patients showed high IFN-γ responses to PPD. In contrary, and for reasons unclear, the 5 subjects that had a positive QFT-GIT but negative TST and negative HBHA-IGRA all had PPD-induced IFN-γ levels below 60 pg/ml.

HBHA-IGRA was repeated at an average frequency of 3 per subject over a median period of 7 months (not repeated in 6 patients and follow-up interrupted in one patient who became pregnant). The majority of patients had stable responses (82%). Four patients had variable test results over time. An additional 3 with an initially negative HBHA-IGRA developed persistently positive tests during follow-up despite no new *Mtb* exposure risk factor. One of these patients had a positive QFT-GIT at baseline (antigen response of 0.82 IU/ml) and another had a favourable response to cART with an increase in CD4^+^ T-cell counts from 457 to 641 cells/mm^3^ and normalization of his CD4^+^/CD8^+^ ratio at the time of test conversion.

### Effect of *Mtb* exposure risk, BCG vaccination status and HIV infection parameters on the LTBI screening test results

A non-random association between baseline test results and selected variables (*Mtb* exposure risk factor, BCG vaccination, CD4^+^ T-cell count and viral load) was investigated. The results are shown in Table 2. A significant association with *Mtb* exposure risk was shown for HBHA-IGRA (p = 0.0353) but not for QFT-GIT. The significance of the association between *Mtb* exposure risk and TST positivity was inconclusive, as the Fisher’s exact test results disagreed with the confidence interval for the odds ratio (a phenomenon that may be caused by the different handling of two-sided inference from asymmetric sampling distributions by these two tests [24]). The same result was found between BCG vaccination and HBHA-IGRA outcome. Furthermore, the variables “BCG vaccination” and “*Mtb* exposure risk factor” were significantly associated (p = 0.0002). A linear trend was present between QFT-GIT and viral load levels (a greater number of positive results occurring in patients with lower viral loads), but no trend was detected between the different screening tests and CD4^+^ T-cell counts.

**Table 2:** **Factors associated with positive LTBI screening test results**

	**TST**		**QFT-GIT**		**HBHA-IGRA**	
	**p**	**OR [CI 95%])**	**p**	**OR [CI 95%]**	**p**	**OR [CI 95%]**
*Mtb* exposure	**0.0455**	11.8 [0.6-232]	0.4455	2.2 [0.5-10.3]	**0.0353**	9.9 [1.1-90.7]
BCG	0.2273	6.0 [0.2-136]	1	0.7 [0.1-5.0]	**0.0318**	15 [0.7-307]
CD4^+^ T-cell count	0.8773	NA	0.7777	NA	0.6021	NA
Viral load	0.1844	NA	**0.0261**	NA	0.0752	NA

A non-random association between positive test results and 1) the presence of an *Mtb* exposure risk factor, 2) BCG vaccination status, 3) CD4^+^ T-cell counts and 4) viral loads was assessed for the TST, the QFT-GIT and the HBHA-IGRA. Fisher’s exact test and odds ratio (OR) with a 95% confidence interval (CI 95%) was applied for the dichotomous variables (presence or not of an *Mtb* exposure risk factor and BCG vaccination status). Chi-squared test for trend was used for CD4^+^ T-cell counts and viral loads organized into ordinal variables (CD4^+^ T-cell counts <50; 50–199; 200–499, >500 cell/mm^3^ and viral loads <40, 40–10000; 10000–100000; >100000 copies/ml). Significant p values are in bold type (p < 0.05).

We next assessed whether HIV-infection parameters (CD4^+^ T-cell counts, CD4^+^ percentages, CD4^+^/CD8^+^ T cell ratios and viral loads) were significant predictor variables of the PPD- and HBHA-induced IFN-γ levels, as shown in Table 3. This was the case for the CD4^+^ T cell percentages and the CD4^+^/CD8^+^ T cell ratios with regards to the levels of PPD-induced IFN-γ. Notably indeterminate results, whether for QFT-GIT or HBHA-IGRA, were not systematically associated to advanced immune-depression, nor extremely high viral loads, as only one of the 5 patients concerned had a CD4^+^ T-cell count <500 cells/mm^3^ (280 cells/mm^3^) and a viral load above 10,000 copies/ml.

**Table 3:** **Effect of HIV on PPD and HBHA induced IFN-γ levels**

	**IFN-γ conc. in response to PPD-stimulation**			**IFN-γ conc. in response to HBHA-stimulation**		
	**n**	**p**	**r**	**n**	**p**	**r**
CD4^+^ T-cell count	43	0.1499	0.2261	42	0.9029	^−^0.0192
CD4^+^ %	42	**0.0034**	0.4473	41	0.5827	0.0873
CD4^+^/CD8^+^ ratio	43	**<0.0001**	0.8586	42	0.4265	0.1245
Viral load	41	0.5777	^−^0.0896	41	0.4643	^−^0.1175

Pearson correlation was used to measure the association between PPD- or HBHA-induced IFN-γ responses and four different predictor variables associated with HIV-infection severity: absolute CD4^+^ T-cell counts, CD4^+^ percentages (%), CD4^+^/CD8^+^ ratios and viral loads. Significant p values are in bold type (p < 0.05). conc = concentration; n = number of tests; r = pearson's product moment coefficient.

### HBHA-IGRA results in patients with active TB

**Figure 2:**
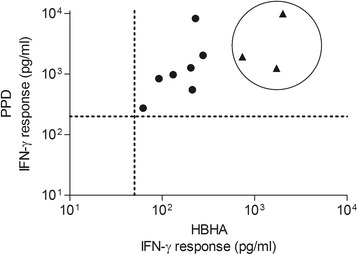
**Comparison of the IFN-γ responses to PPD and HBHA between TB/HIV and LTBI/HIV subjects.** Overall 62 HIV-infected patients were tested with HBHA-IGRA: 48 LTBI screening candidates and 14 patients with clinical suspicion of active TB. At baseline, 10 of these patients had a positive HBHA-IGRA. These 10 positive assays are represented in the graph in terms of the IFN-γ concentrations obtained in response to PPD (Y axis) and HBHA (X-axis) stimulations. The test cut-offs are marked by the dotted lines. The HIV-infected subjects from the LTBI screening group (n = 7) are represented by dots while the HIV patients with confirmed TB are encircled and individually represented by a triangle (n = 3).

Active TB was confirmed for 6 of the 14 HIV-infected patients enrolled with a clinical suspicion of the disease. For these subjects, demographics, HIV parameters and clinical presentation are summarized in Table 4. At baseline, 3 of these patients were HBHA-IGRA responders, all with very high levels of IFN-γ release in response to both PPD and HBHA, as illustrated in Figure 2. HBHA-IGRA was re-tested after at least 1 month of anti-TB treatment (+/− cART) in 4 of the HIV/TB confirmed cases (in 2 HBHA-IGRA responders and 2 non-responders). IFN-γ responses to HBHA persisted for the 2 responders and, at various time points of treatment, converted in the 2 initially non-responders. Two patients developed paradoxal TB-IRIS during treatment but no association was found with baseline HBHA-IGRA response.

**Table 4:** **Characteristics of the HIV subjects with active tuberculosis**

**Subject**	**Demographics**	**Baseline HIV parameters**	**Clinical presentation**
1	47y	CD4: 68 (6%)	Unmasking miliary TB-IRIS
	Caucasian	VL: not tested	
		cART: yes	
2	42y	CD4:124 (14%)	Miliary TB
	Sub saharan	VL: 712000	
		cART: no	
3	28y	CD4: 26 (6%)	Miliary TB
	Sub saharan	VL: 8070000	
		cART: no	
4	38y	CD4: 141 (35%)	Pulmonary TB
	Sub saharan	VL < 40	
		cART: yes	
5	38y	CD4: 25 (5%)	Pulmonary and ganglionary TB
	Sub saharan	VL: 201000	
		cART: no	
6	43y	CD4: 580 (37%)	ART-associated Pleuro-pulmonary TB
	Sub saharan	VL <40	
		cART: yes	

ART: antiretroviral therapy; cART: combination ART; CD4: CD4^+^ T cell count cells/mm^3^ (percentage); TB: Tuberculosis; TB-IRIS: TB-associated immune reconstitution inflammatory syndrome; VL: viral load (copies/ml); y: years.

### Multiplex analysis

**Figure 3:**
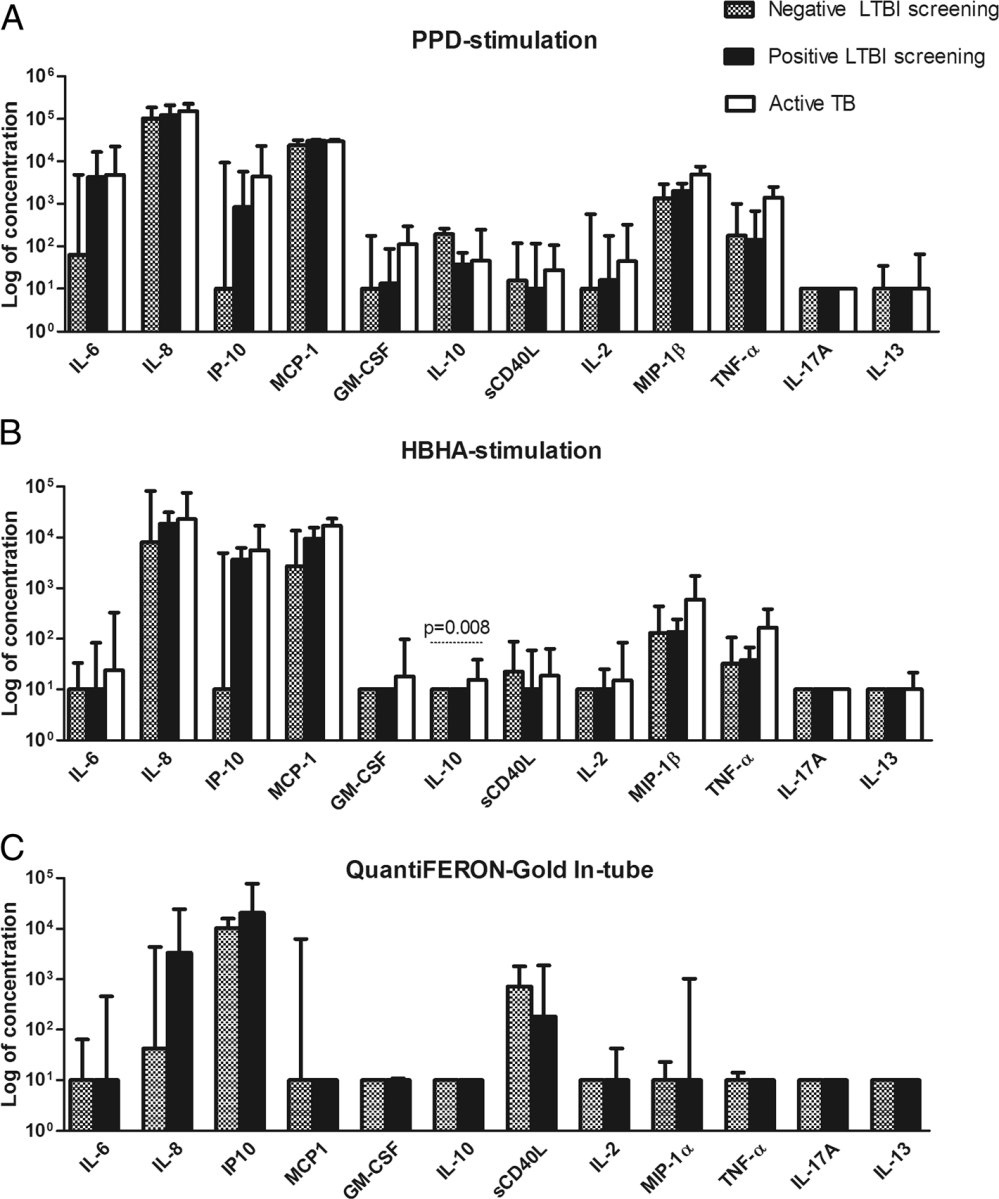
**Multiplex analysis of culture supernatants.** A panel of cytokines and chemokines were measured in the culture supernatants of **(A)** PPD-stimulated peripheral blood mononuclear cells (PBMC), **(B)** HBHA-stimulated PBMC and **(C)** the TB Antigen tube of the QFT-GIT. Median concentrations with interquartile ranges are represented in the graph. Results between subjects with a positive LTBI screening (n = 11), subjects with a negative LTBI screening (n = 8) and active TB patients (n = 6) were compared using Mann–Whitney *U* test or Kruskall-Wallis test. Significant p values are shown.

The cytokine/chemokine profiles of 11 subjects with a positive LTBI screening, 8 subjects with a negative LTBI screening and the 6 confirmed active TB subjects were evaluated and compared. A positive LTBI screening was defined as the presence of at least one positive immunological test (TST, QFT-GIT or HBHA-IGRA), and a negative LTBI screening was defined as all 3 tests being negative and absence of any *Mtb* exposure risk factor. The results are shown in Figure 3. The levels of IL-10 measured in the HBHA-stimulated culture supernatant were statistically higher for the active TB patients when compared with the two other groups (p = 0.008). However, only two of the 6 active TB subjects had IL-10 levels that reached twice the concentrations found in non-stimulated conditions. For these 2 patients, higher analyte concentrations were found for the majority of the cytokines/chemokines measured. The increased level of IL-10 noted in active TB patients was therefore not specific and probably a reflection of the general immune activation induced by the disease.

When we focused on the HBHA-IGRA negative/QFT-GIT positive subjects, IL-6 concentrations exceeded 100 pg/ml in the PPD-stimulated culture supernatants for all 5 concerned subjects. Four of them had IP-10 concentrations above 100 pg/ml in the HBHA-stimulated culture supernatants. However, the increase in sensitivity for the HBHA-IGRA detection of LTBI obtained by the use of these two analytes was offset by a loss in specificity: for both analytes, 3 of the 8 subjects with a negative LTBI screening also showed levels exceeding 100 pg/ml. Likewise, no analyte measured in the QFT-GIT culture supernatants could identify the 3 patients that were only positive for the HBHA-IGRA.

## Discussion

This study is the first to evaluate the use of an HBHA-IGRA as a LTBI screening tool in HIV infected patients living in a low TB incidence country. In this cohort of 48 treatment-naïve HIV-infected subjects, 9 were identified with LTBI when combining a commercialized IGRA (QFT-GIT) and a TST as is currently recommended in Europe [6]. The HBHA-IGRA identified all the patients detected by the TST (including a patient that was negative for QFT-GIT) and recognized a further 3 potential LTBI cases detected by neither the TST nor the QFT-GIT. The HBHA-IGRA was the screening tool that showed the best concordance with *Mtb* exposure risk, as was previously found in a cohort of haemodialysed patients [14]. The test appeared reproducible, as a large majority of patients had stable responses over time, and multiplex analysis confirmed IFN-γ as the best read-out out of 13 chemokines/cytokines. Despite a median follow-up of 14 months, no patients developed active TB. Remarkably, isoniazid prophylactic treatment was prescribed and completed in only one patient after LTBI screening. This illustrates how barriers to LTBI treatment in HIV-infected patients go beyond the problematic of screening tool efficiency, as has been reviewed elsewhere [25,26].

The main limitation of this study is the small number of enrolled patients rendering the results and analysis essentially descriptive. As in all LTBI screening studies, the lack of a gold standard test complicates the interpretation of results. Furthermore, the absence of development of active TB amongst the individuals screened prevents the calculation of the predictive values of the tests. Nonetheless, this pilot study provides support for further larger studies assessing HBHA-IGRA as a complementary tool in the screening for LTBI in HIV-infected patients.

Several studies, both in low- and high-income countries, have evaluated and compared the TST and the commercialized IGRA for LTBI screening in HIV-infected patients, as reviewed by meta-analysis in 2011 [10]. Although the tests appear to have comparable sensitivities, the sensitivity of each test is negatively affected by the immunodeficiency of the patients. Indeed, HIV-infected persons are less likely to have a positive TST or a positive IGRA than HIV-uninfected persons, with test positivity being inversely correlated with the CD4^+^ T-cell count. IGRA indeterminate results are also more frequent in HIV-infected patients when compared with the general population [11]. The T-SPOT.TB®, although inconsistently reported, may have an advantage over QFT-GIT in patients with low CD4^+^ T-cell counts, as the number of PBMC exposed to the mycobacterial antigens in the assay is standardized [9]. This is also the case with the HBHA-IGRA. In this study, a potential negative effect of low CD4^+^ T-cell counts on our in-house assay was not seen. Moreover, during follow-up, only 1 HBHA-IGRA test conversion following favorable immunological response to cART was recorded. However, these results must be interpreted with caution, as the number of subjects enrolled was small and only 2 patients had a baseline CD4^+^ T-cell count below 50 cells/mm^3^.

The HBHA-IGRA, like the commercialized IGRA, offers several advantages over TST including the need of a single visit, less reader bias and the absence of a booster effect on serial testing [11]. The internal negative and positive controls also represent a considerable advantage over TST for which immunological anergy may be incorrectly considered as a negative result. Another essential asset of the commercialized IGRA, QFT-GIT and T-Spot TB®, is the absence of cross-reactivity with BCG vaccination. In the case of HBHA-IGRA, it has been shown that BCG vaccination in infants is capable of priming HBHA specific immune responses [27]. This is conceivable as HBHA is expressed by all members of the *M. tuberculosis* complex including the attenuated BCG strain of *Mycobacterium bovis* from which it is purified. However, it appears that the influence of early-childhood BCG vaccination on HBHA-IGRA becomes negligible in adulthood [13,21,28]. Here the question could not be properly addressed as *Mtb* exposition and BCG vaccination were confounding factors and sample size was too small to perform multivariate analysis.

In line with previous studies, discordant results were found between TST, QFT-GIT and HBHA-IGRA [11,13,14]. These inter-assay discordances persisted even when alternative analytes to IFN-γ were measured in the QFT-GIT and HBHA-IGRA supernatants. It has been suggested that different *Mtb* infection profiles may explain these discrepancies. Indeed, LTBI is now considered as a heterogeneous entity, running from quiescent bacilli to replicating *Mtb* with subclinical disease [29,30]. As the commercialized IGRA use RD-1 antigens that are secreted by actively multiplying *Mtb*, the LTBI patients responding exclusively to these assays are believed to have ongoing *Mtb* replication. In contrary, as T-cell responses to HBHA are considered as markers of latency, HBHA-IGRA exclusive responders are believed to be those controlling *Mtb* replication [31]. In non HIV-infected individuals, it has been suggested that these discordances could allow the classification of LTBI according to the potential of reactivation, a quality that would help target INH prophylaxis [31]. In HIV-infected patients, however, whether the risk of LTBI progression to active TB is different in QFT-GIT and/or HBHA-IGRA responders is unknown. As these patients are at high risk of developing active TB particularly during immune-reconstitution, detecting all those with LTBI appears essential, even those with controlled latency. Combining the QFT-GIT and the HBHA-IGRA could be a suitable option offering a broader detection of the LTBI spectrum. Large-scale studies are necessary to determine whether this combination offers sufficient sensitivity to dismiss TST from LTBI screening in HIV-infected patients, as suggested by our results.

In non HIV-infected patients, the median IFN-γ responses of isolated PBMC to HBHA appear inferior in patients with active TB than in subjects with LTBI, offering a relative discrimination between the two groups [13]. The inferior IFN-γ responses in active TB is due to the presence of regulatory T lymphocytes (Tregs) suppressing T-cell responses to the antigen and to the migration of HBHA-specific lymphocytes to the sites of infected tissue [32,33]. The HBHA-IGRA did not show this for HIV-infected patients, possibly due a high mycobacterial burden or an altered function of Tregs and chemotaxis associated with HIV infection [34,35]. Consequently, like all the currently available LTBI screening tools, the HBHA-IGRA cannot differentiate LTBI from active TB in HIV-infected patients. As HIV-infected patients can develop pauci-symptomatic disease [36], active TB must always be excluded before instauration of prophylactic therapy. When analyzing in greater detail, it appeared that the HBHA-IGRA results in the HIV-positive subjects with active TB were dichotomous: no IFN-γ response was detected in approximately half of the patients, whilst extremely high responses were observed for the others. Factors predicting a HBHA-IGRA response among the HIV/active TB co-infected patients remain unidentified. Elements reflecting or causing altered immune responses, such as CD4^+^ T-cell counts < 200 cells/mm^3^, CD4^+^ percentages < 30%, inversed CD4^+^/CD8^+^ ratios, viral loads >100,000 copies/ml and miliary disease, were found among responders and non-responders. Interestingly, after the instauration of anti-TB therapy (in association or not with cART), the responses to HBHA-IGRA persisted in responders and appeared in subjects with initial negative results. As treated TB subjects are sometimes used as surrogates for LTBI (both representing states of controlled *Mtb* infection), these results underline the HBHA-IGRA’s potential as a LTBI diagnostic tool. Moreover all the patients with TB suspicion that were ultimately disconfirmed, including 2 localized non-tubercular mycobacterial infections, had negative HBHA-IGRA. Overall, in HIV-infected patients with a clinical suspicion of TB, it seems that a very high IFN-γ response to HBHA-IGRA may be considered as an argument in favor of TB diagnosis. However, as for the TST and the commercialized IGRA, a negative result does not exclude active TB. One alternative method that may prove interesting in clinical suspicion of TB may be an HBHA-IGRA performed on cells from the site of disease, rather than peripheral blood mononuclear cells [32], but this approach remains to be investigated in HIV-infected patients.

## Conclusions

This study, although limited in size, offers the first glimpse of the potential diagnostic value of HBHA-IGRA in HIV-infected patients. The assay appears as a complementary tool for LTBI screening, identifying cases not found with the other tests. Future studies should examine whether the combination of QFT-GIT and HBHA-IGRA, which can be performed after a single blood collection, offers sufficient LTBI screening performance to dismiss TST from LTBI screening. In HIV-positive patients, the test could also contribute to the diagnosis work-up of clinical suspicion of TB, as high IFN-γ responses to the assay seem in favour of the diagnosis.
